# How to Efficiently Reduce the Carbon Intensity of the Heavy Industry in China? Using Quantile Regression Approach

**DOI:** 10.3390/ijerph191912865

**Published:** 2022-10-08

**Authors:** Bin Xu

**Affiliations:** School of Management, China Institute for Studies in Energy Policy, Collaborative Innovation Center for Energy Economics and Energy Policy, Xiamen University, Xiamen 361005, China; xubin9675@163.com

**Keywords:** carbon intensity, the heavy industry, quantile regression analysis

## Abstract

This decoupling between carbon dioxide emissions and the heavy industry is one of the main topics of government managers. This paper uses the quantile regression approach to investigate the carbon intensity of China’s heavy industry, based on 2005–2019 panel data. The main findings are as follows: (1) incentive-based environmental regulations have the greater impact on the carbon intensity in Jiangsu, Shandong, Zhejiang, Henan, Liaoning, and Shaanxi, because these provinces invest more in environmental governance and levy higher resource taxes; (2) the impact of mandatory environmental regulations on carbon intensity in Beijing, Tianjin, and Guangdong provinces is smaller, since these three provinces have the fewest enacted environmental laws and rely mainly on market incentives; (3) conversely, foreign direct investment has contributed most to carbon intensity reduction in Tianjin, Beijing, and Guangdong provinces, because these three have attracted more technologically advanced foreign-funded enterprises; (4) technological progress contributes more to the carbon intensity in the low quantile provinces, because these provinces have more patented technologies; (5) the carbon intensity of Shaanxi, Shanxi, and Inner Mongolia provinces is most affected by energy consumption structures because of their over-reliance on highly polluting coal.

## 1. Introduction

Global warming has caused huge damage to the natural ecological environment on which human beings depend [1]; the culprit behind global warming is the accumulation of carbon dioxide. The negative impact of global warming has made all countries realize the necessity of jointly dealing with CO_2_ emissions. The international community has developed a series of agreements to urge countries worldwide to cut CO_2_ emissions in steps, such as the Copenhagen Accord (2009) and the Paris Agreement (2015) [2].

Currently, China’s CO_2_ emissions rank first in the world [3], and statistics show that China’s total of CO_2_ emissions in 2021 was 10.6 billion tons. The heavy industry contributed 55% of China’s CO_2_ emissions during the period 2005–2019. Carbon intensity is a key factor affecting CO_2_ emissions of the heavy industries [4]. The motivation for this article is to investigate the effects of the influencing factors on the carbon intensity of the heavy industries, and to propose targeted countermeasures. The research results can provide empirical support for local governments to formulate industrial policies and can offer new paths for achieving carbon neutrality goals.

The importance of carbon intensity has attracted many scholars to conduct in-depth research into it. Analysis of the existing relevant literature found that these studies have the following two notable characteristics: (1) Existing studies have investigated the impact of environmental regulations on carbon intensity, but they do not break down environmental regulations. These regulations can be further subdivided into mandatory environmental regulations and incentive-based environmental regulations [5]. Moreover, the ways in which these two environmental regulations affect carbon intensity are markedly different; (2) Most literature investigates carbon intensity in the industrial sector using the ordinary least squares. However, a prerequisite for the ordinary least squares to obtain robust parameters is that the sequence of economic variables is normally distributed. In fact, economic phenomena are complex and the data of economic variables have obvious characteristics of “sharp peak” and “thick tail”. This results in the series of economic variables being skewed. Using the ordinary least squares to estimate non-normally distributed variable data will lead to serious adverse consequences, such as increased variance and biased parameter estimators.

The possible contributions of this paper are mainly the following two points: (1) Considering the availability of data, this paper divides environmental regulations into mandatory environmental regulations and incentive-based environmental regulations. This paper investigates the impact of these two environmental regulations on carbon intensity separately. The findings help local governments to flexibly use corresponding environmental policies to promote carbon intensity reduction; (2) Preliminary test results show that the series of economic variables in this paper are not normally distributed. Quantile regression models do not require that the series of economic variables follow a normal distribution. Under the condition that the series of economic variables is not normally distributed, the results of quantile regression are more robust than those of the ordinary least squares [6]. Quantile regression can estimate the effects of influencing factors on carbon intensity at different levels, including maximum, minimum and median values. The ordinary least squares can only give an average effect of influencing factors on carbon intensity. Thus, the research results can provide empirical support for local governments to formulate effective emission reduction policies.

Apart from the introduction, this paper has the following sections: the literature review is placed in Section 2; the theoretical model and the construction of the empirical model are placed in Section 3; the empirical results are placed in Section 4; the discussion section is placed in Section 5; the results of the robustness test are presented in Section 6; policy recommendations are placed in Section 7; and the Limitation of the study and future recommendations are placed in the last section.

## 2. Literature Review

This section selects several representative influencing factors of carbon intensity for comment. China is now the world’s largest CO_2_ emitter; in order to achieve low-carbon growth, the Chinese government has taken many measures to control fossil energy use and reduce CO_2_ emissions, such as expanding investment in environmental governance and promulgating environmental laws. Most of these measures fall into the category of environmental regulations. Therefore, this section first reviews the existing literature on environmental regulations.

(1) Mandatory environmental regulations. In a survey in China, Liang et al. [7] found that mandatory environmental regulations promoted the improvement of environmental efficiency and the decrease in carbon intensity. The scale of China’s manufacturing industry was the largest in the world, and its carbon intensity was high [8]. Jiang et al. [9] pointed out that mandatory environmental regulations had not played a role in improving energy-saving technologies and reducing carbon intensity. However, Wang and Zhu [10] reached the opposite conclusion. Using a spatial econometric model, they found that mandatory environmental regulations helped to reduce carbon intensity. The main reason was that mandatory environmental regulations prompted heavy industrial enterprises to move from the provinces with strict environmental regulations to the provinces with low environmental regulations. A difference-in-difference model survey indicated that mandatory environmental regulations were beneficial for reducing the carbon intensity of the heavy industry [11]. The main reason was that mandatory environmental regulations increased the pressure on industrial enterprises, prompting industrial enterprises to change environmental strategies [12].

Compared with mandatory environmental regulations, incentive-based environmental regulations were flexible. Therefore, incentive-based environmental regulations could play a better role in environmental governance.

(2) Incentive-based environmental regulations. Incentive-based environmental regulations include many means of environmental governance, such as resource tax, carbon tax, carbon emission permits, and wastewater discharge permits [13]. However, the main task of developing countries was to facilitate the rapid economic development and social employment [14]. Therefore, many developing countries implemented loose incentive-based environmental regulations [15]. This attracted many investment projects in the heavy industries, such as the petrochemical, plastics processing, and metallurgy industries [16]. The lax incentive-based environmental regulations had made many heavy industrial companies pay little attention to improving energy efficiency and had resulted in high carbon intensity [17]. During the 12th and 13th Five-Year Plans, China’s incentive-based environmental regulations had continuously strengthened and played a critical role in reducing carbon intensity [18]. Using a stochastic frontier approach, Yao et al. [19] found that incentive-based environmental regulations had prompted industrial enterprises to increase investment in technology research and development. Technological innovation could not only decrease the use of fossil energy but also expand the application of abatement equipment, thereby contributing to the reduction of carbon intensity.

Environmental regulations were mainly formulated by the governments. Energy prices were not only affected by domestic factors, but also by international factors. The heavy industry was energy-intensive and sensitive to energy prices. Soaring oil prices would prompt industrial enterprises to expand clean energy use, helping to reduce carbon intensity. The existing literature on the relationship between energy prices and carbon intensity is reviewed below.

(3) Energy prices. The production activities of the heavy industries often required a large amount of energy input [20]. Rising fossil energy prices turned to increase cost for heavy industry producers [21]. To reduce production costs and enhance market competitiveness, heavy industry production companies had begun to focus on improving energy efficiency and reducing carbon intensity [22]. Natural gas was a clean energy source, and many countries strongly encouraged natural gas consumption [23]. Government departments had expanded financial subsidies that had lowered natural gas prices. The decline in clean energy prices incentivized heavy industry producers to gradually increase the use of clean energy, thereby promoting carbon emission reductions and mitigating carbon intensity [24]. In recent years, major energy-consuming countries have accelerated the development of wind power and photovoltaic power [25]. Governments have also provided financial subsidies for wind power and photovoltaic power generation, thereby reducing renewable energy prices [26]. This induced heavy industry to expand the use of clean electricity, contributing to CO_2_ emission reduction and carbon intensity mitigation.

Energy prices could affect the energy structure of the heavy industries. A sharp drop in oil prices would help increase the share of fossil fuels in energy structure, resulting in a high carbon intensity. A literature review of the relationship between energy structure and carbon intensity is given below.

(4) Energy structure. In general, the heavy industry still relied mainly on highly polluting coal to meet energy needs [27]. Coal had a high carbon content, as its excessive use resulted in the emission of carbon dioxide and high carbon intensity [28]. Using comparative analysis, Rojas-Cardenas et al. [29] examined the carbon intensity in Mexico and the world’s energy-consuming countries; they showed that the carbon intensity of Mexico’s steel industry was low, since the main source of energy consumption was clean natural gas. Similarly, Switzerland’s heavy industrial production plants increased biomass energy use and significantly reduced the share of coal in energy consumption [30]. The carbon intensity of China’s heavy industrial sector had historically been high, mainly because many heavy industries still relied on high-emission coal [31]. A coal-dominated energy mix remained a major obstacle to cutting down carbon intensity [32].

In the long run, technological progress was an important factor of carbon intensity. More low-carbon technologies would certainly help mitigate carbon intensity. The literature on the relationship between technological progress and carbon intensity was described as follows.

(5) Technological progress. Employing the decomposition approach, Song et al. [33] studied the heavy industry and showed that technological advances could help reduce carbon intensity. Moreover, the level of technology had obvious regional heterogeneity. It was because there were significant regional differences in R&D funding and technological talent [34]. This made the contribution of technological innovation to carbon intensity in different regions significantly different [35]. Findings from the Swedish steel industry showed that updating furnace technology and equipment could significantly improve energy efficiency and reduce coal use, thereby contributing to the mitigation of carbon intensity [36]. The European steel industry analysis also found that updating blast furnace combustion technology and carbon capture technology could significantly reduce carbon intensity [37].

Technological progress could help industrial enterprises to update energy utilization technology and reduce energy intensity, thereby contributing to the reduction of carbon intensity. The literature on the relationship between energy intensity and carbon intensity is reviewed below.

(6) Energy intensity. The heavy industry was an energy-intensive industry, as its energy intensity was significantly higher than that in other industries such as the light industries, services, and agriculture [38]. Under the background of China’s energy consumption mainly relying on highly polluting fossil energy, high energy intensity made the reduction in carbon intensity a difficult task [39]. Shandong and Liaoning provinces were two major industrial provinces in China, where many heavy industrial enterprises gathered [40]. Therefore, reducing energy intensity was key in facilitating the decline of carbon intensity. A survey of the heavy industries in India yielded similar findings that energy intensity was one of the key factors influencing the changes in carbon intensity [41].

Reviewing the above research literature, it is found that most of the sources use mean models to analyze the impact of environmental regulations and other determinants on carbon intensity. The obtained research results have little reference value for local governments to manage the environment and reduce carbon intensity. Therefore, this paper first subdivides environmental regulations into incentive-based environmental regulations and mandatory environmental regulations. Quantile regression methods were then used to investigate the impact of these two environmental regulations and other determinants on carbon intensity. The quantile regression can estimate the heterogeneous impact of the influencing factors on carbon intensity, including the effect of the maximum, minimum, and median values.

## 3. Method and Model Specification

### 3.1. The Basic Principle of Quantile Regression

Economic and financial variable series are often characterized by spikes and thick tails. The strength of the influence of the independent variable on the maximum, minimum, and intermediate values of the dependent variable may vary significantly. The traditional mean models (e.g., linear panel data model, and distributed lag model) can only obtain the average influence of the explanatory variables on the dependent variable. For example, carbon intensity varies from province to province, and the effects of explanatory variables on the provinces with high carbon intensity are different from those with low carbon intensity.

Quantile regression can overcome the shortcomings of the mean models. The advantages of quantile regression models are as follows: (1) Quantile regression does not require the series of economic variables to follow a normal distribution. Economic phenomena are volatile, so the series of economic variables are often non-normally distributed. When the series of economic variables is not normally distributed, the estimated results of quantile regression are more robust than those of the mean models; (2) Quantile regression can give the comprehensive influence of the explanatory variables on the dependent variables, including the influence of maximum, minimum and median values [42]. Thus, the quantile regression has obvious advantages over traditional mean models. The mathematical formula of quantile regression approach is shown in Equations (1) and (2) below:

(1) $$y_{i}=x_{i}^{\prime }\beta_{\theta }+\mu_{\theta i},0<\theta <1$$

(2) $$Quant_{\theta }(y_{i}|x_{i})=x_{i}\beta_{\theta }$$

where the vector of explanatory variables is signified by *X*, the dependent variable is signified by *Y*, Quantile regression is expressed by quant. The specific theory of quantile regression can refer to Koenker and Hallock [43]. This paper uses the bootstrap method to estimate the parameters of the quantile regression model. The specific estimation process of the bootstrap method can refer to Efron [44].

### 3.2. Theoretical Mechanism and Model Specification

According to the status of China’s heavy industry and literature review, this paper selects the main influencing factors of carbon intensity. The theoretical mechanism between carbon intensity and influencing factors is explained as follows:

(1) Energy structure. The heavy industry is highly energy-intensive. Although many heavy industrial enterprises are continuously expanding the use of clean energy, coal still occupies a large proportion of energy structure in the heavy industry [45]. The carbon intensity of coal is high, and the coal-dominated energy mix makes it difficult to reduce carbon intensity. Drawing on the experience of existing research, this paper applies the rate of coal consumption to measure energy structure (%);

(2) Technological progress. Technological innovation and application are important means of solving environmental problems. Technological progress will help break through the key core technologies of the heavy industry and improve energy efficiency [46]. In addition, technological progress is conducive to the large-scale application of new clean energy such as shale gas extraction, wind power, and photovoltaic power generation. Advances in cleaner production technology help reduce CO_2_ emissions in the production process or end-of-line treatment. From the perspective of production scale, technological progress can help improve the marginal productivity of factors, drive enterprises to expand output, and make up for the increase in cost. Neither productivity improvements nor the use of carbon abatement technologies can help cut down the carbon intensity of the industrial sector. This paper adopts the ratio of technology R&D input to GDP to represent the variable value of technological progress (%);

(3) Incentive-based environmental regulations. Environmental pollution is mainly caused by industrial production activities such as CO_2_ emissions and smog pollution. In order to reduce environmental pollution, government departments must play a regulatory role. Incentive-based environmental regulations are a means for government departments to control environmental pollution through market means, such as technological R&D, carbon emission permit system, and carbon emission trading markets [47]. There are two theories about the relationship between incentive-based environmental regulations and carbon intensity. The first theory holds that incentive-based environmental regulations will increase the production cost of enterprises, divert entrepreneurial energy, and affect the profitability of enterprises. This restricts industrial enterprises from expanding investment in technology research and development, and low technology levels are not conducive to reducing carbon intensity. The second theory holds that incentive-based environmental regulations promote enterprise innovation, which in turn reduces energy consumption and improves product quality. Therefore, incentive-based environmental regulations can help reduce CO_2_ emissions and mitigate carbon intensity;

(4) Mandatory environmental regulations. Mandatory environmental regulations are used to control environmental pollution in the form of laws or rules: (1) Stringent environmental regulations have a major impact on CO_2_ emissions and carbon intensity. High-polluting industrial enterprises often face serious consequences such as high financial penalties, production stoppages, and factory closures. This caused a huge panic in the heavily polluting enterprises, which reduces the expected rate of return and restricts the expansion of reproduction. It is not conducive to improving corporate energy efficiency and reducing carbon intensity; (2) Mandatory environmental regulations require industrial enterprises to disclose environmental information through the Environmental Protection Law. As a result, environmental information transparency of industrial enterprises has been improved. Information disclosure reduces the asymmetry of environmental information between industrial enterprises and relevant stakeholders and strengthens external supervision. This forces industrial companies to focus on energy consumption and CO_2_ emissions, which in turn contributes to a reduction in carbon intensity;

(5) Foreign direct investment. China’s heavy industry is changing from an extensive growth mode to an intensive growth mode. To achieve green and sustainable growth, the Chinese government has continuously strengthened foreign investment reviews. Introduced investment projects often have advanced technology and management models [48]. Expanding foreign direct investment can attract advanced manufacturing companies from developed countries into the Chinese market. The advanced management experience and production technology of foreign-funded enterprises have spillover effects. Thus, it can lead to an improvement in technologies, and promote a decline in carbon intensity among domestic heavy industry enterprises. Foreign direct investment is measured by the amount of foreign investment actually introduced (100 million yuan (CNY));

(6) Oil prices. International oil price fluctuations are normal. If oil prices rise sharply, the heavy industry expands the use of alternative energy sources, such as clean gas and biofuels [49]. Conversely, the decline in international oil prices will prompt energy-intensive heavy industry companies to expand oil use. Over-reliance on carbon-rich oil leads to high carbon intensity in the industrial sector. The oil trading volume of the West Texas, Brent, and Dubai oil markets has accounted for the largest proportion of the total oil transactions. This paper calculates the average price of oil transactions in these three oil markets, and uses it to measure the international oil price (CNY/ton);

(7) Economic growth. The economic growth of many countries has experienced a path from extensive growth to intensive growth. After high-input, high-output growth in the early stages, the Chinese government has realized that extensive growth is unsustainable. On the one hand, the Chinese government actively promotes the high-tech industries such as high-end equipment manufacturing. On the other hand, the government strictly controls the scale of heavy industry. The development of these industries can improve technologies and expand the supply of renewable energy, thereby promoting mitigation in carbon intensity [50].

The above theoretical analysis has shown that the selected socio-economic factors closely relate to carbon intensity. Therefore, this paper takes these factors into the analytical framework and constructs an empirical model of carbon intensity (Equation (3)).

(3) $$\begin{array}{l} LCI_{it}=C+\beta_{1}LENS_{it}+\beta_{2}LTEC_{it}+\beta_{3}LIER_{it}+\beta_{4}LMER_{it}\\ +\beta_{5}LFDI_{it}+\beta_{6}LPRI_{it}+\beta_{7}LGDP_{it}+\mu_{it} \end{array}$$

where *L* means that the variable data is logarithmically processed. *ENS* means energy structure, the carbon intensity is measured by *CI*, technological progress is represented by *TEC*, incentive-based environmental regulations are represented by *IER*, mandatory environmental regulations are represented by *MER*, *FDI* means foreign direct investment, *PRI* means oil prices, and *GDP* means economic growth. The constant term is delegated by *C*, and the disturbance term is delegated by *μ*. The specific form of quantile regression is shown in Equation (4).

(4) $$\begin{array}{l} Q_{\tau }(LCI_{it})=C_{\tau }+\beta_{1\tau }LENS_{it}+\beta_{2\tau }LTEC_{it}+\beta_{3\tau }LIER_{it}\\ +\beta_{4\tau }LMER_{it}+\beta_{5\tau }LFDI_{it}+\beta_{6\tau }LPRI_{it}+\beta_{7\tau }LGDP_{it}+\mu_{it} \end{array}$$

where *τ* signifies the quantile point. This paper selects 5 representative quantiles, which are 10th, 25th, 50th, 75th, and 90th.

### 3.3. Variable Selection and Data Source

The sample in this paper is the annual panel data of China’s 30 provinces from 2005 to 2019 (Table S1). (1) Explained variable: Carbon intensity of the heavy industry (10,000 t/100 million CNY). Carbon intensity = the heavy industry’s CO_2_ emissions/the output of the heavy industry. CO_2_ emissions are obtained by multiplying different types of fossil energy by their carbon emission coefficients and then adding them up. Emission output is distinct from carbon intensity. Emission output = the heavy industry’s output/the heavy industry’s CO_2_ emissions. It can be seen that emission output is equivalent to a reciprocal of carbon intensity. Much literature has used carbon intensity to measure the low-carbon level of industrial development or economic growth [51,52]. Drawing on the existing literature, this paper uses carbon intensity to measure the carbon level of the heavy industry.

(2) Explanatory variables: First, the energy consumption structure is represented by the proportion of coal consumption (%). Second, technological progress is expressed by the ratio of technological innovation funding to GDP (%). Third, Foreign direct investment is expressed in the amount of foreign capital introduced (100 million CNY). Fourth, oil prices. The representative international oil markets mainly include the Dubai Petroleum Exchange Market in the UAE, the Brent Petroleum Exchange Market in the United Kingdom, and the West Texas Petroleum Exchange Market in the United States. This article uses the average oil prices of the three major oil markets as the variable value of oil prices (CNY/ton). Fifth, incentive-based environmental regulations are delegated by the ratio of investment in environmental governance to GDP (%). Sixth, this paper uses the number of environmental laws enacted by the government to represent mandatory environmental regulations (Piece). Seventh, economic growth. GDP per capita can objectively measure the level of regional economic development. Carbon intensity, economic growth, oil prices, and foreign direct investment have been deflated to remove the effects of inflation. The raw data for the economic variables come from the Wind database (https://www.wind.com.cn/, (accessed on 1 January 2021)). The definitions and units of the variables used are presented in Table 1. The statistical results of the variable series are presented in Table 2.

## 4. Results

### 4.1. Unit Root Test

Since the 1960s, the development of econometrics has entered a fast lane. Econometric methods have become mainstream economics, sociology, and management research methods. However, the premise of econometric model regression is that the variable series is stationary. The unit root test is generally used to judge whether an economic series is stationary. If the socio-economic variable sequence has unit roots, it becomes a non-stationary sequence. Many existing studies have proved that a unit root process in an economic sequence implies non-stationarity and that a pseudo-regression problem in regression analysis may occur. The unit root test has now become an indispensable step in econometric analysis. If the probability value of the statistic value of the unit root test is less than 10%, it means that the tested economic variable sequence is stationary. On the contrary, it means that the series of variables is non-stationary. This paper uses the IPS, ADF, ADF–Fisher, Breitung test, Fisher-PP, and CIPS tests to examine the economic series used. Different from other testing methods, the CIPS test can determine whether the series of economic variables is stationary on the basis of considering the possible cross-sectional correlation. In order to improve the reliability of the test results, this paper uses these six methods to perform unit root tests. The formula for the main tests is listed in Appendix A. The results in Table 3 show that all economic series are not stationary.

### 4.2. Cointegration Tests

Cointegration theory was first proposed by Engle and Granger [53]. It lays a theoretical foundation for finding equilibrium relations between two or more non-stationary variables and establishing error correction models with cointegration relations. Cointegration is the sequence when multiple economic variables show as non-stationarity, but a specific linear combination of these variables shows stability. Under these circumstances, a long-term stable relationship can exist between these variables. The methods suitable for testing the cointegration relationship of panel data mainly include the Pedroni test and the Kao test. Based on the logarithmic variable data, this paper uses these two methods to perform cointegration tests (Table 4). The probability values of the statistical values in Table 4 are all less than 10%, indicating that there is a causal nexus between carbon intensity and its determinants.

### 4.3. Multicollinearity Test

Econometric theory states that multicollinearity problems will arise if the explanatory variables are closely related. Multicollinearity includes perfect multicollinearity and approximate multicollinearity. Perfect multicollinearity will have some serious consequences, such as non-existence of parameter estimates and infinite variance of parameter estimates. Approximate multicollinearity produces undesirable results as follows: (1) the variance of the parameter estimates increases; (2) the confidence intervals of the parameter estimates become larger; and (3) the t-test of the estimated parameters may not be significant. The methods of multicollinearity test mainly include: (1) simple correlation coefficient method; (2) variance inflation factor (VIF) method; and (3) Klein’s discriminant rule. The VIF method has the advantages of easy operation and objective test process. Therefore, this paper uses the VIF method to perform a multicollinearity test. The formula of VIF is as follows:

(5) $$VIF_{j}=\frac{1}{\left(1-R_{j}^{2}\right)}$$

where $R_{j}^{2}$ represents the goodness of fit. *J* indicates that the *j*-th explanatory variable is used as the dependent variable for regression estimation. If *VIF_j_* ≥ 10, it indicates there is a severe multicollinearity between the explanatory variable and other explanatory variables. Moreover, this multicollinearity has serious adverse effects on the results of the ordinary least squares estimation. Conversely, if *VIF_j_* is less than 10, it indicates that the multicollinearity is weak and will not have a serious adverse effect on the estimation results. The results in Table 5 show that all the values of VIF are less than 10, indicating that model 4 does not have severe multicollinearity.

### 4.4. Tests of Normal Distribution

**Figure 1:**
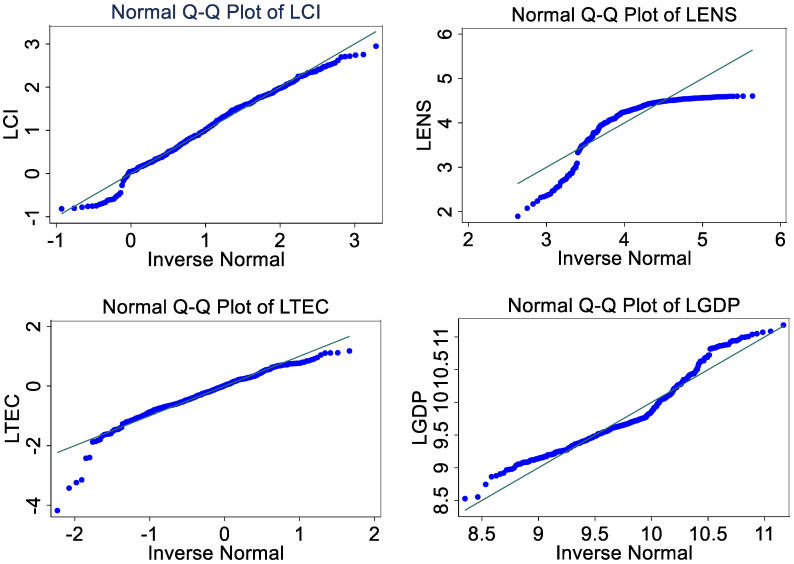

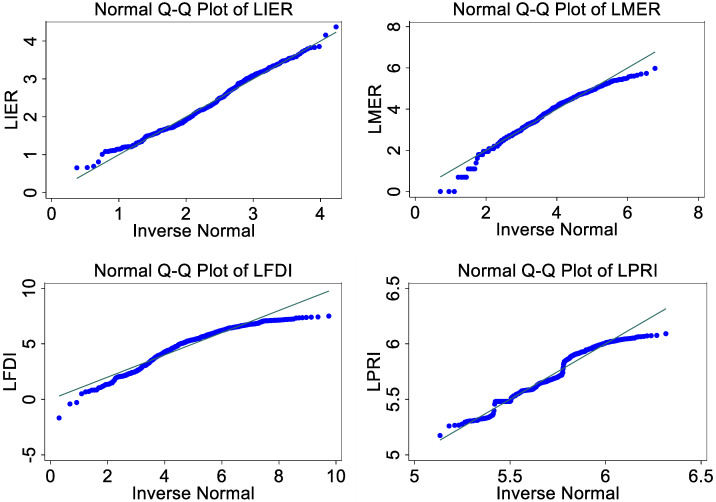
Q-Q diagram of economic variable series.

For traditional econometric models, an important prerequisite for obtaining good estimates is that the variable sequence obeys a normal distribution. However, many research results have shown that the series of economic variables is often non-normally distributed. Under the condition that the economic series is not normally distributed, the result of quantile regression is more robust. Therefore, before the model regression, this paper uses the Q-Q diagram (i.e., quartile–quartile) to test whether the economic series used is normally distributed. The results in Figure 1 show that the blue variable curve does not completely fit the X = Y line, which indicates that these economic series are not normally distributed. In this case, it is more reasonable to use quantile regression to investigate carbon intensity.

### 4.5. Quantile Regression Results

**Figure 2:**
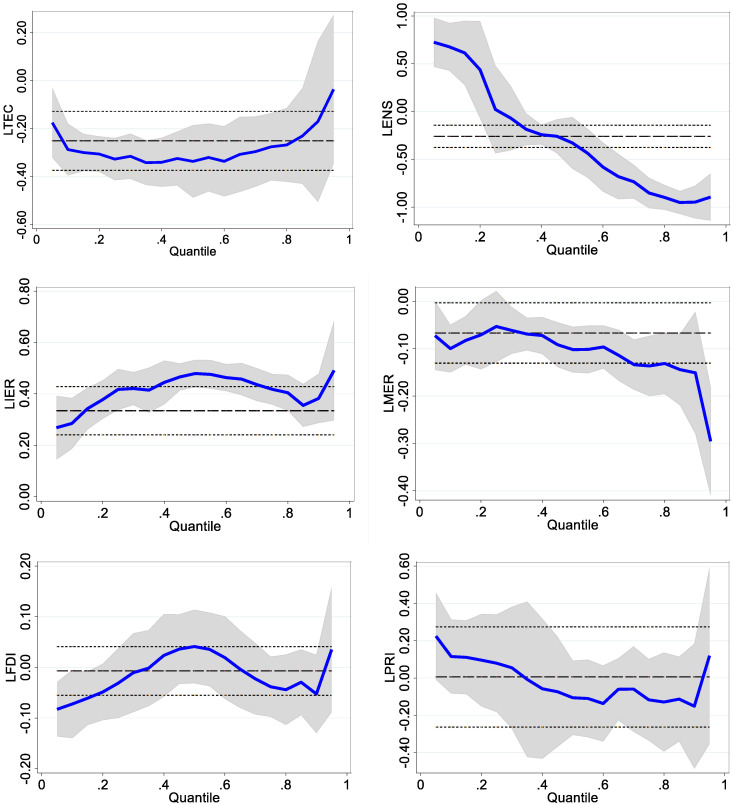

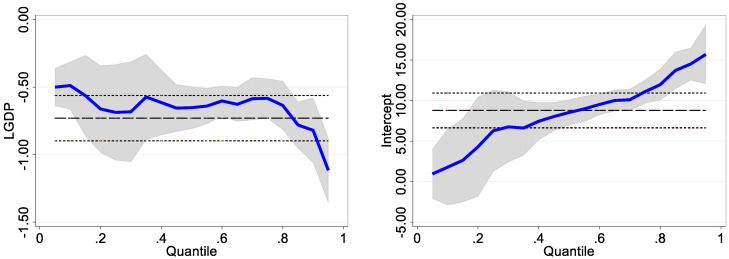
Plot of quantile estimates of carbon intensity.

According to the level of carbon intensity, this paper divides the 30 administrative units into 6 groups (Table 6). The results of quantile regression are listed in Table 7, and the graph of quantile regression results is placed in Figure 2. In addition, this paper also uses the mean models for regression, and compares its results with that of quantile regression (Table 7):

(1) Incentive-based environmental regulations. The carbon intensity in the 10th–25th, 25th–50th, and 50th–75th quantile groups receives a larger impact from incentive-based environmental regulations, with coefficients of 0.418, 0.479 and 0.418, respectively. These quantile groups mainly include Jiangsu, Shandong, Fujian, Hunan, Zhejiang, Henan, Hebei, Guangxi, Liaoning, Hebei, and Shanxi provinces. These provinces are home to many industrial enterprises, such as coal processing plants, petrochemical, iron and steel, and cement enterprises. To achieve low-carbon growth, local governments strengthen environmental regulations. Strict environmental regulations have prompted industrial companies to upgrade technology and equipment, which further cut down carbon intensity;

(2) Mandatory environmental regulations. Carbon intensity receives a negative effect from mandatory environmental regulations, as its regression coefficients ranging from the lower 10th quantile group to the upper 90th quantile group are −0.010, −0.053, −0.102, −0.136, and −0.151, respectively. A negative coefficient indicates that increasing the formulation and implementation of environmental laws has the effect of reducing carbon intensity. Furthermore, mandatory environmental regulations have a greater impact on carbon intensity in the 25th–50th, 50th–75th, and 75th–90th quantile groups. This is mainly because these provinces have enacted more environmental laws to deal with industrial pollution;

(3) The regression coefficients of energy consumption structure in the 25th–50th, 50th–75th, and 75th–90th quantile groups are negative, −0.328, −0.852, and −0.948, respectively. This result implies that changes in energy structure have contributed to the decline in carbon intensity. This is because the rate of clean energy in total energy consumption continues to expand, which is conducive to reducing CO_2_ emissions. These groups include Yunnan, Sichuan, Guangxi, Shanxi, Shaanxi, Inner Mongolia, Liaoning and Jilin. There are many rivers in Yunnan, Sichuan, and Guangxi, and the vertical drop of the rivers is large. The central government and local governments continued to increase hydropower development, and their hydropower output increased rapidly. Local industrial enterprises expand the use of hydropower, which improves energy structure and further drives down carbon intensity. Shanxi, Shaanxi and Inner Mongolia are major coal producing provinces. The local government encourages coal-to-gas production. Local industrial companies expand gas use, which helps reduce carbon intensity;

(4) The parameter estimates of technological progress in all quantile groups are negative, namely −0.287, −0.327, −0.336, −0.275, and −0.170, respectively. This means that technological innovation is conducive to reducing carbon intensity. Technology is an important contributor to achieving carbon intensity reduction. In recent years, economic development has enabled government finance and industrial enterprises to allocate more funds to scientific and technological innovation. The number of granted patents has grown rapidly, and the application of patented technology helps industrial companies reduce carbon intensity;

(5) Foreign direct investment is negatively related to carbon intensity, which means that expanding foreign investment can contribute to the decline of carbon intensity. To improve the technical level of industrial enterprises, the government vigorously introduces foreign investment projects with advanced technology and less pollution. These technologically advanced and low-energy-consuming projects have driven domestic industrial enterprises to upgrade their technologies and equipment, reducing carbon intensity;

(6) The parametric estimates of oil prices are negative in the high quantile groups, indicating that oil prices have contributed to the decline in carbon intensity. International oil prices have generally risen, although with occasional sharp fluctuations. This brings a heavy economic burden to local industrial enterprises and is not conducive to the long-term stable development of this industry. Therefore, local governments actively develop clean energy and accelerate the replacement of oil resources. Expanded use of clean energy reduces CO_2_ emissions, thereby contributing to a reduction in carbon intensity;

(7) Economic growth. The regression coefficients of economic growth in all groups are negative, −0.489, −0.688, −0.652, −0.584, and −0.820, respectively. This denotes that economic growth is contributing to a reduction in carbon intensity. The coefficient values are all between −1 and 0. It indicates that for every 1% increase in economic growth, carbon intensity decreases by less than 1%. The decline in carbon intensity is less than the increase in economic growth, and the main reasons for this are as follows: the mode of economic growth is undergoing a transition from an extensive economic growth to green economic growth; governments at all levels vigorously develop the high-tech industries and tertiary industries, and strictly control the scale of heavy industry; technological innovation and equipment renewal brought about by the development of high-tech industries are applied in the heavy industries, which promotes the reduction in carbon intensity; and economic growth still emits carbon dioxide, but the carbon intensity of economic growth is gradually decreasing.

## 5. Robustness Test

To test the reliability of the estimated results of carbon intensity, this paper adopts the method of changing variables’ values to test the robustness. In Section 4.5, the value of foreign direct investment (FDI) is measured by total foreign direct investment, and technological progress is measured by the share of R&D investment in GDP. This section uses FDI per capita as the variable value of FDI and uses the number of patents per 10,000 people to measure technological progress. Then the paper uses the quantile regression model for regression estimation. The results in Table 7 show that the signs of the regression coefficients for foreign direct investment (LFDI) and technological progress (LTEC) are consistent with the results in Table 6. Combining the results in Table 7 and Table 8, it can be seen that the quantile regression has high robustness, and the estimation results are reliable.

## 6. Discussion

Several of the above empirical results deserve further discussion.

### 6.1. The Carbon Intensity in the 10th–25th, 25th–50th, and 50th–75th Quantile Groups Are More Affected by Incentive-Based Environmental Regulations

This result differs from the conclusion of Yang et al. [54]. Using a nonlinear mediating effect model, they found a positive U-shaped nonlinear relationship between environmental regulations and carbon intensity. The provincial differences in environmental governance investment and resource tax can explain this result. (1) Environmental governance investment. An important feature of the incentive-based environmental regulations is the economic leverage it carries out when influencing pollutant emissions of industrial enterprises. Government departments affect the decision-making of industrial enterprises via the utilization of prices, taxes, charges, subsidies, credits, etc. The implementation of incentive environmental policies leads to an increase in the production cost of industrial enterprises. The larger the energy consumption scale of the enterprise, the greater the cost. This forces industrial companies to expand technological research and equipment upgrades, thus promoting a decline in carbon intensity. Statistics show that from 2005 to 2019 the average investment in industrial pollution control in the 10th–25th, 25th–50th, and 50th–75th quantile groups was 4.04 billion CNY, 1.90 billion CNY, and 1.85 billion CNY, respectively, which was higher than that in the lower 10th quantile group (1.77 billion CNY), the 75th–90th quantile group (1.43 billion CNY) and the upper 90th quantile group (1.14 billion CNY). More pollution control investment can not only update production technology, but also help to expand the use of carbon abatement equipment. The 10th–25th, 25th–50th, and 50th–75th quantile groups have more investment in environmental governance. This helps industrial enterprises to carry out technological innovation and mitigate carbon intensity.

(2) Resource tax. The resource tax turns the cost of environmental pollution into the internal cost for industrial enterprises. The resource tax is flexible and can effectively give full play to the subjective initiative of enterprises and promote their green transformation [55]. An example is the government environmental protection agency’s levy resource tax on coal mining companies. The imposition of resource tax has led to rising coal prices and increased production costs for industrial enterprises. Governments are concerned about economic development and strengthening environmental supervision. Many heavy industrial enterprises are distributed in the 10th–25th, 25th–50th, and 50th–75th quantile provinces, such as Henan, Hebei, Shandong, Liaoning, Shaanxi, Hubei, Shanxi, and Guizhou. The formulated resource tax controls the serious environmental pollution and CO2 emissions caused by industrial production. The resource tax levied by the above quantile provinces is more. This urges local industrial enterprises to update technology and equipment; therefore, the carbon intensity of the 10th–25th, 25th–50th, and 50th–75th quantile provinces is affected more by incentive environmental regulation.

### 6.2. Mandatory Environmental Regulations Significantly Impact Carbon Intensity in the Lower 10th Quantile Group

This result differs from the findings of Hou et al. [56]. Using a dynamic threshold effect model, they investigated China’s industrial sector and found that environmental regulations affect carbon intensity through industrial structure transformation. Strict environmental regulations are not conducive to reducing carbon intensity. The provincial differences in the number of environmental decrees could explain the result in this paper. Mandatory environmental regulations are a direct regulatory tool, and an important means to achieve the purpose of environmental governance and are an administrative-led environmental governance mechanism. Under the direct regulation mode, the government relies on its power to directly regulate energy use and pollution emissions. In the process of economic construction, the role of the government is not only a “night watchman”, but an “active participant”. The Chinese government participates in environmental governance by formulating relevant environmental laws and regulations. In general, mandatory environmental regulations are more restrictive to industrial enterprises. If the pollutant discharge of industrial enterprises exceeds the standard, there will be serious consequences such as severe administrative penalties, fines, suspension of production activities or business closures [57]. An analysis of the statistics found that from 2005 to 2019, the governments in the lower 10th quantile provinces enacted the smallest environmental laws, with an average of 42 environmental laws per year. Other provinces have enacted more environmental ordinances. From the 10th–25th quantile group to the upper 90th quantile group, the average environmental regulations were 112, 78, 63, 51, and 46, respectively. The lower 10th quantile group includes Beijing, Tianjin, and Guangdong provinces; these three provinces are economically developed and have strong economic strength. Local government departments prefer to use incentive-based environmental regulations to promote industrial enterprises to upgrade technology and reduce CO_2_ emissions, such as clean energy subsidies and R&D capital investment. Therefore, the contribution of imperative environmental regulations to the carbon intensity in the lower 10th quantile group is relatively small.

### 6.3. Technological Progress Contributes More to the Carbon Intensity in the Lower 10th, 10th–25th, and 25th–50th Quantile Groups

This result differs from the findings of Gu et al. [58], who do not give provincial differences in the impact of technological progress on carbon intensity. The results of this paper are mainly attributable to the obvious differences in the number of patents and energy intensity. (1) Provincial differences in the number of patents. For a long time, the heavy industries have been high energy-intensive, and their main source of energy consumption is high-emission coal. In order to control CO_2_ emissions, governments at all levels and industrial enterprises have increased investment in research and development. Statistics show that from 2005 to 2019 the average growth rate of government financial investment in science and technology reached 15.0%. The growth rate of technological research and development expenditures of industrial enterprises was 18.4%. Rapidly increasing R&D investment helps to obtain more patented technologies, such as energy-saving, carbon emission reduction, carbon capture, and renewable energy technologies. The statistics show that from 2005 to 2019 the average numbers of patents granted in the lower 10th, 10th–25th, and 25th–50th quantile groups were 158,146 pieces, 140,986 pieces, and 54,871 pieces, respectively. However, the number of patents granted in the 50th–75th, 75th–90th, and upper 90th quantile groups in these provinces was 32,672 pieces, 24,130 pieces, and 35,954 pieces. The number of patents granted in the lower quantile provinces was higher. The lower 10th, 10th–25th, and 25th–50th quantile groups have more advanced patented technologies, such as high-efficiency and energy-saving melting technology, and blast furnace energy-saving equipment. The promotion of many advanced patented technologies helps to reduce coal use and control CO_2_ emission growth, thereby contributing to the reduction in carbon intensity. Therefore, technological progress contributes more to the decline in carbon intensity in these quantile groups.

(2) Provincial differences in energy intensity. Energy intensity is an important path through which technological progress affects carbon intensity [59]. Technological progress helps industrial companies use more advanced energy-efficient equipment, thereby reducing energy intensity. Coal and oil have long been the main sources of heavy industrial enterprises. Low energy intensity helps reduce fossil energy use, further contributing to a reduction in carbon intensity. Statistics show that from 2005 to 2019, the energy intensity of the lower 10th quantile provinces, 10th–25th quantile provinces, and 25th–50th quantile provinces were the lowest, at 0.39 ton/10,000 CNY, 0.74 ton/10,000 CNY, and 1.04 ton/10,000 CNY, respectively. However, the energy intensity of 50th–75th quantile provinces, 75th–90th quantile provinces, and upper 90th quantile provinces was 1.81 ton/10,000 CNY, 2.34 ton/10,000 CNY, respectively. Low energy intensity means that the same output can be obtained with less energy consumption. The energy intensity of the lower 10th quantile provinces, 10th–25th quantile provinces, and 25th–50th quantile provinces is the lowest, resulting in technological progress having the greatest impact on carbon intensity.

### 6.4. Foreign Direct Investment Has a Larger Impact on Energy Intensity in the Lower 10th Quantile Group

This result differs from the findings of Cai et al. [60]. They divide foreign direct investment into outward foreign direct investment (OFDI) and inward foreign direct investment (IFDI), finding that OFDI leads to an increase in carbon intensity and IFDI helps reduce carbon intensity. The differences in foreign direct investment can explain this result in this paper. The “pollution shelter hypothesis” holds that foreign direct investment will cause polluting industries to migrate to developing countries due to the loosening of environmental regulations, and this will exacerbate environmental pollution in developing countries. Since the economic reforms in 1980, China has been one of the most active host countries for foreign direct investment inflows. Foreign direct investment activates the market economy, promotes social employment, and drives Chinese domestic industrial enterprises to focus on technological innovation. While China has become the “processing factory of the world,” it also produces many CO_2_ emissions. In recent years, Chinese governments have strictly monitored foreign-funded projects and vigorously introduced foreign-funded projects with advanced technology [61]. Through technology spillovers, foreign direct investment drives Chinese domestic industrial enterprises to upgrade their technologies and equipment, promoting a reduction in carbon intensity. Statistics show that from 2005 to 2019, the average foreign direct investment in the lower 10th quantile group was 380.7 billion CNY, much more than that in the 10th–25th quantile group (268.0 billion CNY), the 25th–50th quantile group (73.3 billion CNY), the 50th–75th quantile group (56.4 billion CNY), the 75th–90th quantile group (36.2 billion CNY), and the upper 90th quantile group (176.1 billion CNY). The lower 10th quantile group brings in the most investment, making a foreign direct investment the largest contributor to the reduction in carbon intensity.

### 6.5. Energy Consumption Structure Significantly Reduces the Carbon Intensity in the 50th–75th, 75th–90th, and Upper 90th Quantile Groups

The results of this paper show that the energy consumption structure has a positive impact on the carbon intensity of some quantile provinces and exerts a negative impact in other provinces. This result differs from the findings of Yu et al. [62], who found that the improvement of energy consumption structure is helpful to reduce carbon intensity. The provincial differences in coal consumption can explain the above results in this paper. China’s coal reserves are abundant and easy to exploit. The open-pit coal mines are distributed in many provinces in China, such as Inner Mongolia, Shanxi and Shaanxi provinces. Heavy industry production requires much energy, and to ensure adequate energy supply most of the heavy industries get situated in areas rich in coal resources or areas with convenient shipping, such as Anshan City in Liaoning Province, Panzhihua City in Sichuan Province, Shanghai City, and Huainan in Anhui province. For a long time, heavy industry enterprises have mainly used coal for production activities. The heavy use of coal has made the heavy industry the number one source of CO_2_ emissions, which results in carbon intensity being high [63]. Reducing coal use and increasing clean energy consumption can significantly reduce carbon intensity. For example, many local governments have expanded energy subsidies to support natural gas and biomass use. The implementation of these measures has gradually reduced the proportion of coal. Many provinces in the 50th–75th, 75th–90th, and upper 90th quantile groups have numerous heavy industrial enterprises, such as Liaoning, Shanxi, Shanxi, Jilin, Heilongjiang, and Shaanxi provinces. These provinces are large coal-producing areas, providing sufficient energy sources to develop the heavy industry. In the context of carbon emission reduction targets, these provinces have significantly reduced direct coal combustion and increased the use of clean energy. For example, Shanxi, Shaanxi, and Inner Mongolia provinces vigorously developed a coal-to-gas technology and expanded the production scale of coal-to-liquids gas. The energy structure has been significantly improved so that the energy consumption structure contributes more to reducing carbon intensity.

## 7. Conclusions and Policy Implications

### 7.1. Conclusions

After performing the normality test, this paper investigates carbon intensity using a quantile regression model. The results obtained are as follows: (1) the carbon intensity in the 10th–25th, 25th–50th, and 50th–75th quantile groups is more affected by incentive-based environmental regulations, because the provinces in these groups invest more environmental governance funds and collect more resource taxes; (2) mandatory environmental regulations played a significant role in reducing carbon intensity and contributed the most to the decline in carbon intensity in the lower 10th quantile group; (3) technological progress contributes more to the carbon intensity in the low quantile provinces such as Guangdong, Beijing, Tianjin, Fujian, and Hainan; (4) foreign direct investment has a larger impact on energy intensity in Tianjin, Beijing, and Guangdong provinces, because these provinces bring in more foreign-funded enterprises; and (5) the energy structure played a role in significantly reducing the carbon intensity in the high quantile groups.

### 7.2. Policy Implications

#### 7.2.1. The Lower 10th, 75th–90th, and Upper 90th Quantile Groups Should Improve the Environmental Tax System and Expand Environmental Governance Input

The coefficients of incentive-based environmental regulations in all quantile groups are all positive, indicating that incentive-based environmental regulations have not played a significant role in reducing carbon intensity. Therefore, all quantile groups should further increase environmental governance investment and raise the standard of environmental tax. (1) The governments could formulate corresponding pollution tax standards based on each region’s economic development. The standard of pollutant discharge fees should be higher than the income from enterprises stealing pollutants. This can prompt manufacturers to pay attention to pollution taxes and take specific actions to reduce CO_2_ emissions, such as biofuels and pure electric vehicles. In addition, the environmental protection department could further improve the environmental monitoring system and strictly review the pollution discharge of all enterprises to ensure that the environmental protection tax is fully collected. (2) The government expands channels and increases pollution control investment. Fiscal funds for pollution control are often insufficient, which leads to unsatisfactory industrial pollution control. Government departments can adopt flexible mechanisms to encourage private capital to enter the field of environmental governance. For example, the government encourages private enterprises to participate in the construction of industrial parks, professionally deal with industrial CO_2_ emissions, and obtain corresponding economic income. (3) Local governments should give full play to the leverage of financial funds and use limited financial funds to leverage more social capital into the fields of pollution control and environmental protection. This can give play to the multiplier effect of financial funds and achieve the impact of attracting more investment with the least amount of funds. Taking into account the main role of enterprises in pollution prevention and control, the government should guide enterprises to increase investment in environmental protection. For example, the tax department can lower the tax rate to encourage enterprises to increase investment in environmental protection equipment and improve production technology.

#### 7.2.2. The Lower 10th Quantile Group Can Strengthen the Supervision of the Implementation of Mandatory Environmental Regulations

The coefficients of mandatory environmental regulations in all quantile groups are negative, indicating that the implementation of environmental laws has played a significant role in reducing carbon intensity. However, the absolute value of the coefficient for the lower 10th quantile group is the smallest, indicating that mandatory environmental regulations contribute the least to the reduction in carbon intensity. Therefore, the local government should further strengthen the formulation of environmental laws and strict environmental assessment systems. (1) In implementing command-type environmental regulations, the government should focus on implementing policies flexibly. It can focus on opening up more market areas and increasing market competition, forcing heavily polluting enterprises to transform faster. The government can formulate cleaner production demonstration projects and green technology promotion catalogs. Industrial enterprises with demonstration projects can enjoy a certain percentage of tax relief and financial subsidies. (2) The central government should improve the performance appraisal system and strictly assess the main responsibility of local governments for environmental protection. Local governments can establish a dynamic assessment mechanism for regional environmental quality and increase the weight of environmental protection in the assessment of local officials. During the promotion process of officials, the government adds the step of evaluating environmental performance. The government should bundle environmental protection with the “political life” of officials, which can ensure the continuity of local environmental protection goals.

#### 7.2.3. The Government Should Take Various Measures to Increase Investment in Technology R&D

The absolute value of the coefficient of technological progress in the 50th–75th, 75th–90th, upper 90th quantile groups is smaller than that in the lower 10th, 10th–25th and 25th–50th quantile groups. It indicates that the contribution of technological progress to reducing the carbon intensity of the 50th–75th, 75th–90th, upper 90th quantile groups is relatively small. Therefore, these quantile groups should increase technological personnel training and research and development investment to improve the technical level. (1) The government should provide credit guarantees to encourage financial institutions to inject funds into technological innovation. Technological innovation activities are high risk and high investment, and many companies do not have sufficient funds for technological innovation. Therefore, the financial support of financial institutions is crucial. The government provides guarantees for financial loans, which can ensure that R&D institutions receive much-needed funds. In addition, the government has formulated preferential policies to facilitate the entry of technology-based enterprises into the financial market. This has helped tech companies tap into the bond and stock markets and be well funded. (2) The government should increase support for the research and development of low-carbon technology. Firstly, the government can improve the scientific research platform and increase the guidance of research and development policies. For example, the government establishes a platform for enterprises and research institutes to communicate with each other. This helps research institutions to promote the acquired low-carbon technologies to the market as soon as possible. Second, the taxation department collects environmental protection tax and uses a certain percentage of the tax to subsidize green technology innovation. This can improve the enthusiasm of enterprises to engage in technology research and development and encourages industrial enterprises to update emission-reduction equipment. (3) The government should formulate differentiated carbon emission reduction targets and paths. Resource-based provinces can accelerate the transformation of the “extensive” development mode to the “high-efficiency, clean, and low-carbon” mode. The government accelerates the optimization of energy structure and promotes the transformation of technological progress to a low-carbon direction. Non-resource-based provinces should vigorously promote the development of energy technology and develop high-tech industries. Accelerating the optimization of the industrial structure can improve energy efficiency and promote carbon emission reduction in the heavy industry.

#### 7.2.4. The 10th–25th, 25th–50th, 50th–75th, 75th–90th, and Upper 90th Quantile Groups Are Supposed to Strengthen the Management of Foreign-Funded Projects

The impact strength of foreign direct investment on carbon intensity in the 10th–25th, 25th–50th, 50th–75th, 75th–90th, and upper 90th quantile groups is low. Therefore, these quantile groups should take various measures to introduce more high-tech projects. (1) Local governments should reject the introduction of energy-intensive projects such as steel projects, petrochemicals, and non-ferrous metal processing projects. For heavy industrial projects that need to be introduced urgently, the government can control them through carbon taxes, carbon permits, and carbon emission trading. This helps control heavy industry scale, remove excess capacity, and improve energy efficiency. (2) The government should further adjust and optimize the structure of foreign direct investment and introduce foreign direct investment with low-carbon and new energy technologies. The government should formulate policies to give full play to the technology spillover effect of foreign direct investment to improve the low-carbon technology of domestic industrial enterprises. For example, the government can encourage foreign-funded enterprises to merge or acquire small and medium-sized industrial enterprises. This will help improve the technology and equipment level of the overall industrial sector as soon as possible and reduce energy consumption and carbon intensity. (3) The government should encourage domestic companies to establish joint ventures with foreign companies. This can expand the introduction of foreign capital and enable domestic enterprises to master advanced management models and technologies as soon as possible. Furthermore, the central government encourages the central and western regions to expand the introduction of foreign capital. (4) In the process of introducing foreign capital, local governments had better pay attention to the different CO_2_ emissions in different industries. The government can increase investment in higher education and research institutes to improve technology absorptive capacity. This can give full play to the technological spillover effect of foreign direct investment and drive industrial enterprises to update emission reduction technologies.

#### 7.2.5. The Lower 10th, and 10th–25th Quantile Groups Should Adopt Incentives to Urge Heavy Industry Producers to Expand the Use of Low-Carbon Energy

The coefficients of energy consumption structure in the lower 10th, and 10th–25th quantile groups are positive numbers (i.e., 0.677 and 0.022), indicating that energy consumption structure is not conducive to reducing carbon intensity. Therefore, these quantile groups should actively promote industrial enterprises to expand the use of clean energy. (1) The local governments should encourage heavy industry producers to expand the use of coal gas. China has abundant coal resources and under the conditions that the coal-to-gas technology has matured, the government can encourage coal gas production and adopt an energy subsidy policy to encourage heavy industry producers to expand the use of coal gas. (2) The government ought to mobilize various resources to increase natural gas extraction and imports. For example, the government should adjust policies to allow private enterprises to extract natural gas. It will speed up natural gas extraction and increase natural gas production. Meanwhile, government departments should increase natural gas imports by sea and land. On the one hand, the western region should expand natural gas cooperation with Central Asian countries and expand natural gas imports. This can meet the natural gas consumption needs of heavy industrial enterprises in the central and western regions. On the other hand, the eastern coastal areas should increase their natural gas import by sea routes, such as Australia, Indonesia, and Qatar. (3) The government should accelerate the development of natural gas-fired power generation and mobilize the enthusiasm of natural gas production enterprises, power generation enterprises and power grid enterprises. The government expands the laying of natural gas transmission pipelines in areas where industrial enterprises gather and expands the scope of application of natural gas in the industrial sector. The financial department provides financial support to encourage industrial enterprises to update equipment, improve the efficiency of natural gas use, and increase the enthusiasm of implementing “from coal to natural gas”. Local governments should rationally plan the distribution of industrial parks and the sites of renewable energy production enterprises. The western region is rich in solar energy and wind power resources. The local governments can build industrial parks near photovoltaic production bases to conveniently deliver the clean electricity produced to industrial enterprises.

## 8. Limitation of the Study and Future Recommendation

### 8.1. Limitation of the Study

This paper uses the panel quantile regression model to investigate the carbon intensity of China’s heavy industry, and the estimated results show the heterogeneous effects of influencing factors on the carbon intensity across quantile provinces. However, the quantile regression model is still a linear regression model. Existing research results show that the relationship between economic variables is more likely to be nonlinear; this is because economic phenomena are complex and changeable. The quantile regression model cannot estimate the possible non-linear relationship between the explanatory variable and the dependent variable. This is a shortcoming of this article.

### 8.2. Future Recommendation

The nonparametric econometric models are data-driven. In nonparametric econometric models, the relationship between the explanatory variable and the explained variable is determined by the variable data itself, and there is no artificial setting. Therefore, the nonparametric econometric model can realistically simulate the real relationship between the influencing factors and carbon intensity. In the future, we will construct a new nonparametric econometric model and use it to examine the carbon intensity of China’s heavy industry. The research results can provide empirical support for the government to formulate targeted energy policies according to different development stages.

## Figures and Tables

**Table 1:** Definition and unit of variables in this paper.

Variable	Definition	Unit
CI	Carbon intensity	Ton/10^4^ CNY
ENS	Energy consumption structure	%
TEC	Technological progress	%
IER	Incentive-based environmental regulations	%
MER	Mandatory environmental regulations	Piece
FDI	Foreign direct investment	100 million CNY
PRI	Oil prices	CNY/ton
GDP	Economic growth	CNY

**Table 2:** The statistical description of variables.

Variable	Units	Mean	Std.dev.	Min	Max	Obs
CI	Ton/10^4^ CNY	4.17	2.98	0.44	19.05	450
ENS	%	69.23	23.87	6.67	99.90	450
TEC	%	0.92	0.56	0.02	3.24	450
IER	%	15.75	12.12	2.22	84.70	450
MER	Piece	66.68	61.49	1.0	394.0	450
FDI	100 million CNY	446.0	478.7	0.31	2247.7	450
PRI	CNY/ton	387.0	84.2	240.0	527.4	450
GDP	CNY	42,638	27,018	5052	164,220	450

Notes: Obs represents observation.

**Table 3:** Results of unit root tests.

Series	LLC	Breit	PP	IPS	ADF	CIPS	Obs
LCI	−9.86 ***	0.47	226.41 ***	−4.97 ***	128.88 ***	−2.345	450
LENS	−0.86	2.13	80.81 **	−6.93 ***	65.34	−2.887 **	450
LTEC	0.47	5.18	19.96	5.11	29.40	−2.337	450
LIER	−5.23 ***	−0.19	73.58	−1.92 **	78.65 *	−1.941	450
LMER	−3.74 ***	4.16	100.62 ***	0.47	72.85	−3.058 ***	450
LFDI	−0.81	3.92	62.22	0.44	71.21	−2.058	450
LPRI	−0.92	−2.69 ***	68.67	−1.44 *	58.28	−2.003	450
LGDP	−1.97 **	6.11	22.00	4.71	24.18	−1.484	450

Notes: The above results of the test, under the condition that the constant and trend terms are included. At the 10% significance level, passing a significant test is indicated by *; At the 5% significance level, passing a significant test is indicated by **; At the 1% significance level, passing a significant test is indicated by ***. The above results are implemented by Stata software.

**Table 4:** Result of panel cointegration test.

Method	Statistics	Statistical Value (*p*-Value)
Kao test	Augmented Dickey–Fuller	2.395 ***
	Unadjusted Modified Dickey–Fuller	−1.700 **
Pedroni test	Modified Phillips–Perron	8.443 ***
	Phillips–Perron	−10.436 ***
	Augmented Dickey–Fuller	−9.505 ***

Notes: The above results are implemented by Stata software. ** and *** indicate the significance levels of 5% and 1%, respectively.

**Table 5:** Results of the variance inflation factor (VIF).

Explained Variable	R^2^	VIF	Judgement Result
LENS	0.229	1.297	˂10
LTEC	0.583	2.398	˂10
LIER	0.266	1.362	˂10
LMER	0.358	1.558	˂10
LFDI	0.357	1.555	˂10
LPRI	0.126	1.144	˂10
LGDP	0.572	2.336	˂10

**Table 6:** Grouped results of 30 provinces according to the level of carbon intensity.

Quantile Group	Provinces
lower 10th	Tianjin, Beijing, Guangdong
10th–25th	Jiangsu, Shandong, Fujian, Jiangxi
25th–50th	Hunan, Zhejiang, Henan, Hainan, Guangxi, Chongqing, Hebei, Gansu
50th–75th	Anhui, Yunnan, Liaoning, Shaanxi, Hubei, Xinjiang, Guizhou, Shanxi
75th–90th	Qinghai, Jilin, Inner Mongolia, Sichuan
upper 90th	Shanghai, Ningxia, Heilongjiang

**Table 7:** Quantile regression result.

Variables	Quantile Regression					Median
	10th Quant	25th Quant	50th Quant	75th Quant	90th Quant	
Constant	1.786	6.270 ***	8.529 ***	11.094 ***	14.501 ***	8.529 ***
LENS	0.677 ***	0.022	−0.328 **	−0.852 ***	−0.948 ***	−0.328 ***
LTEC	−0.287 ***	−0.327 ***	−0.336 ***	−0.275 ***	−0.170 **	−0.336 ***
LIER	0.285 ***	0.418 ***	0.479 ***	0.418 ***	0.383 ***	0.479 ***
LMER	−0.010 ***	−0.053 *	−0.102 ***	−0.136 ***	−0.151 **	−0.102 ***
LFDI	−0.072 ***	−0.031 **	0.041 *	−0.038 ***	−0.052 **	−0.041 **
LPRI	0.115 **	0.079 ***	−0.106 **	−0.117 **	−0.151 *	−0.106 *
LGDP	−0.489 ***	−0.688 ***	−0.652 ***	−0.584 ***	−0.820 ***	−0.652 ***
Pseudo R^2^	0.390	0.313	0.316	0.309	0.306	0.316

Notes: Median regression is represented by median. *, ** and *** indicate the significance levels of 10%, 5% and 1%, respectively.

**Table 8:** Robustness test: the results of quantile estimation.

Variables	Quantile Regression					Median
	10th Quant	25th Quant	50th Quant	75th Quant	90th Quant	
Constant	1.915	5.120 **	7.724 ***	9.799 ***	15.237 ***	7.724 ***
LENS	0.337 ***	0.078	−0.358 *	−0.889 ***	−0.866 ***	−0.358 ***
LTEC	−0.307 ***	−0.289 ***	−0.204 ***	−0.187 ***	−0.054	−0.204 ***
LIER	0.189 *	0.258 ***	0.313 ***	0.395 ***	0.367 ***	0.313 ***
LMER	−0.033	−0.054	−0.083 ***	−0.113 ***	−0.205 ***	−0.083*
LFDI	−0.107 ***	−0.086 **	0.028	−0.048	−0.043	−0.028
LPRI	0.340 **	0.287 *	−0.287 **	−0.173	−0.084	−0.287
LGDP	−0.001	−0.170	−0.336 ***	−0.353 ***	−0.994 ***	−0.336 **
Pseudo R^2^	0.423	0.341	0.327	0.321	0.304	0.428
Obs	450	450	450	450	450	450

Notes: *, ** and *** indicate the significance levels of 10%, 5% and 1%, respectively.

## Data Availability

Readers can request the original data of the article by email.

## Supplementary Materials

The following supporting information can be downloaded at: https://www.mdpi.com/article/10.3390/ijerph191912865/s1, Table S1: The original data of “How to efficiently reduce the carbon intensity of the heavy industry in China? using quantile regression approach”.

## Appendix A

Panel unit root test methods mainly include IPS (Im–Pesaran–Shin), Fisher-ADF, LLC (Levin–Lin–Chu), and Breitung tests.

(1) The equation of the LLC test is as follows:
  (A1) $$\Delta y_{n,t}=\alpha y_{n,t-1}+\sum_{i=1}^{P}a_{n,i}\Delta y_{n,t-1}+d_{n,t}^{\prime }b_{n}+e_{nt}$$
  where *a* is the autoregressive coefficient, and *p* is the lag order.
(2) Breitung test. Assuming that ∆*y*_nt_ and *y_n_*_,*t*−1_ are regressed on their lag term (∆*y_n_*_,*t*−1_, ∆*y_n_*_,*t*−2_,…, *∆y_n_*_,*t*−*p*_), the residuals obtained are *e*_1,*nt*_ and *e*_2,*nt*_, respectively. Then, the residuals are subjected to orthogonal dispersion transformation, and the following formula is obtained:
  (A2) $$e_{nt}^{*}={\left(\frac{T-t}{T-t+1}\right)}^{1/2}(e_{nt}^{**}-\frac{1}{T-t}\sum_{l=t+1}^{T}e_{nt}^{*})$$
  Finally, perform the following regression:
  (A3) $$e_{nt}^{*}=\alpha e_{nt}^{**}+r_{nt}$$
(3) The equation of IPS test is as follows:
  (A4) $$\Delta y_{nt}=\alpha_{n}y_{n,t-1}+\sum_{t=1}^{P}a_{nt}\Delta y_{n,t-1}+d_{nt}^{\prime }b_{n}+v_{nt},t=p_{n}+2,p_{n}+3,\cdots ,T$$
(4) The assumptions of the Fisher-ADF test are as follows:
  (A5) $$H_{0}:\alpha_{n}=0\begin{array}{cc} & n=1,2,\cdots ,N \end{array}H_{1}:\left\{\begin{array}{l} \alpha_{n}<0n=1,2,\cdots ,N_{0}\\ \alpha_{n}=0n=N_{0}+1,N_{0}+2,\cdots ,N \end{array}\right.$$

The formula for the test statistic is as follows:

(A6) $$\bar{t}=\frac{1}{N}\sum_{1}^{N}t_{n}$$

Each test method has unique characteristics and a different range of applications. The ADF test is suitable for testing economic series with higher order autoregression.
